# Genetic Characterization of the Tetracycline-Resistance Gene *tet*(X) Carried by Two *Epilithonimonas* Strains Isolated from Farmed Diseased Rainbow Trout, *Oncorhynchus mykiss* in Chile

**DOI:** 10.3390/antibiotics10091051

**Published:** 2021-08-29

**Authors:** Christopher Concha, Claudio D. Miranda, Javier Santander, Marilyn C. Roberts

**Affiliations:** 1 Laboratorio de Patobiología Acuática, Departamento de Acuicultura, Universidad Católica del Norte, Coquimbo 1780000, Chile; christopher.concha@ucn.cl; 2 Centro AquaPacífico, Coquimbo 1780000, Chile; 3 Marine Microbial Pathogenesis and Vaccinology Laboratory, Department of Ocean Sciences, Memorial University of Newfoundland, St. John’s, NL A1C 5S7, Canada; jsantander@mun.ca; 4 Department of Environmental and Occupational Health Sciences, School of Public Health, University of Washington, 4225 Roosevelt Way NE, Suit #100, Seattle, WA 98105, USA; marilynr@u.washington.edu

**Keywords:** *tet*(X), *Epilithonimonas*, salmon farming, fish pathogen, tetracycline resistance, aquaculture, Chile

## Abstract

The main objective of this study was to characterize the *tet*(X) genes, which encode a monooxygenase that catalyzes the degradation of tetracycline antibiotics, carried by the resistant strains FP105 and FP233-J200, using whole-genome sequencing analysis. The isolates were recovered from fin lesion and kidney samples of diseased rainbow trout *Oncorhynchus mykiss*, during two Flavobacteriosis outbreaks occurring in freshwater farms located in Southern Chile. The strains were identified as *Epilithonimonas* spp. by using biochemical tests and by genome comparison analysis using the PATRIC bioinformatics platform and exhibited a minimum inhibitory concentration (MIC) of oxytetracycline of 128 µg/mL. The *tet*(X) genes were located on small contigs of the FP105 and FP233-J200 genomes. The sequences obtained for the *tet*(X) genes and their genetic environment were compared with the genomes available in the GenBank database of strains of the *Chryseobacterium* clade belonging to the *Flavobacterium* family, isolated from fish and carrying the *tet*(X) gene. The Tet(X) proteins synthesized by the Chilean *Epilithonimonas* strains showed a high amino acid similarity (range from 84% to 100%), with the available sequences found in strains belonging to the genus *Chryseobacterium* and *Flavobacterium* isolated from fish. An identical neighborhood of *tet*(X) genes from both Chilean strains was observed. The genetic environment of *tet*(X) observed in the two strains of *Epilithonimonas* studied was characterized by the upstream location of a sequence encoding a hypothetical protein and a downstream located alpha/beta hydrolase-encoding gene, similar to the observed in some of the *tet*(X) genes carried by *Chryseobacterium* and *Flavobacterium* strains isolated from fish, but the produced proteins exhibited a low amino acid identity (25–27%) when compared to these synthesized by the Chilean strains. This study reports for the first time the carriage of the *tet*(X) gene by the *Epilithonimonas* genus and their detection in fish pathogenic bacteria isolated from farmed salmonids in Chile, thus limiting the use of therapies based on oxytetracycline, the antimicrobial most widely used in Chilean freshwater salmonid farming. This results suggest that pathogenic strains of the *Chryseobacterium* clade occurring in Chilean salmonid farms may serve as important reservoirs of *tet*(X) genes.

## 1. Introduction

In Chilean salmon freshwater farms, the high prevalence of bacterial infections, such as Flavobacteriosis mainly caused by the pathogen *Flavobacterium psychrophilum*, has stimulated the use of important amounts of antimicrobials [1,2,3]. In 2020, the Chilean salmonid farming industry used 379.6 tons to produce a biomass of 1,075,896 tons of harvested fish, of which 2.44% was used in freshwater Chilean salmonid farms [4]. Oxytetracycline was the most used antimicrobial in freshwater Chilean salmonid farming during 2020, accounting for 79.52% of antimicrobials administered in this environment [4], and from these, 38.26% was used for the treatment of Flavobacteriosis episodes, in which external signs of disease are commonly treated through a bath with oxytetracycline [2].

Aquaculture settings have been suggested as environments with a high diversity of *tet* genes, encoding for tetracycline resistance [5,6], perhaps due to the wide use of oxytetracycline to treat fish diseases [7]. Thus, the persistence and enrichment of *tet* genes in the aquaculture environments can be significantly enhanced by the administration of oxytetracycline-based therapies, prompting the need of a continuous surveillance.

Previously, several studies demonstrated an important occurrence of oxytetracycline-resistant bacteria in Chilean land- and lake-based farms associated with Chilean freshwater farming [8,9]. It have previously been reported the carriage of several *tet* genes, such as *tet*(A), *tet*(B), *tet*(E), *tet*(H), *tet*(L), *tet*(34), *tet*(35), and *tet*(39) by various bacterial species recovered from various Chilean aquaculture sources, including reared fish, pelletized feed, and water and sediment from lake-based farms [10,11]. In a more recent study, the encoding-resistance genes *tet*(A), *tet*(B), *tet*(C), *tet*(L), *tet*(M), *tet*(S), *tet*(W), and *tet*(X) were reported in various points in riverine waters located upstream and downstream from the discharge of effluents of various freshwater rainbow trout (*Oncorhynchus mykiss*) rearing farms in Chile [7]. As noted in the article, the most common disease occurring in the five studied trout farms was Flavobacteriosis causing a 3–4% mortality in each farm.

Most of the known *tet* genes confer resistance to tetracyclines by encoding for efflux proteins (33 genes), whereas a smaller number of *tet* genes conferring resistance to tetracyclines by encoding ribosomal protection proteins (12 genes) or for enzymes that chemically modify tetracycline (13 genes) have been currently reported (http://faculty.washington.edu/marilynr/, accessed on 10 August 2021).

The *tet*(X) gene encodes a NADP-dependent monooxygenase that catalyzes the degradation of tetracycline antibiotics, including tigecycline [12,13,14]. It is well-known that *tet*(X)-carrying bacteria exhibit high levels of resistance to all clinical important tetracyclines [15], but until now, no studies reporting the occurrence of this gene in bacteria isolated from Chilean salmonid farms are available. In only one study it was demonstrated the occurrence of *tet*(X) genes in pathogenic fish bacteria belonging to the *Flavobacterium* family, identified as *Chryseobacterium balustinum* (identity: 95.5%) and recovered from rainbow trout in the UK [16].

*Epilithonimonas* is a bacterial genus belonging to the *Chryseobacterium* clade, thus closely related to the bacterial genus *Chryseobacterium*, the second largest genus in the *Flavobacteriaceae* family [17,18]. *Epilithonimonas* sp. FP211-J200 is the first representative of this genus associated with fish diseases [19], but *tet*(X) genes have never been previously reported in this genus. The main aim of the study was to characterize *tet*(X) genes and their genetic background carried by two *Epilithonimonas* strains isolated from diseased fishes from two Chilean freshwater salmonid farms.

## 2. Results

### 2.1. Phenotypic Characterization

**Figure 1:**
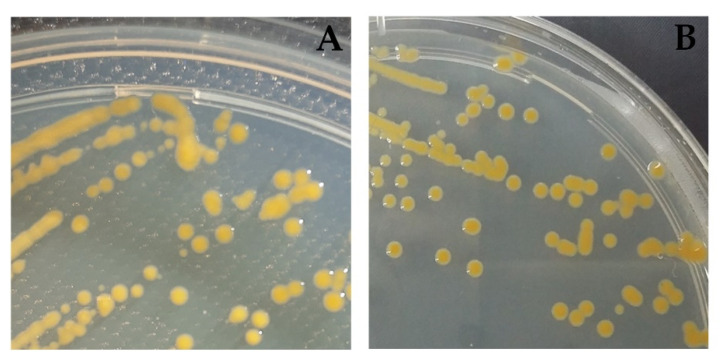
Colony morphotypes of the *Epilithonimonas* strains recovered from diseased rainbow trout from Chilean farms grown on TYES agar: (**A**) FP105; (**B**) FP211-J200.

The bacterial strains FP105 and FP211-J200 showed the phenotypic characteristics typical of members of the *Chryseobacterium* clade belonging to the *Flavobacterium* family. Strains FP105 and FP211-J200 were found to be Gram-negative, rod-shaped, non-motile, positive for production of oxidase, indole and catalase, reduction of nitrate to nitrite and hydrolysis of aesculin, negative for acid production from glucose and able to form flexirubin-type pigments. When grown on TYES agar, the colonies were circular, smooth, convex and bright orange with diameters from 1.5 to 2.5 mm (Figure 1). Both strains were able to grow on R2A and Tryptone Soy agar plates, but not on MacConkey agar. Growth of strains occurred at 15 and 28 °C, but not at 37 and 42 °C, and cannot tolerate 2% NaCl.

The API ZYM profiles of the FP105 and FP211-J200 strains are presented in Table 1, showing the capacity of both isolates to produce the enzymes alkaline phosphatase, esterase (C4), lipase (C8), leucine arylamidase, valine arylamidase, acid phosphatase, and naphthol-AS-BI-phosphohydrolase, whereas only FP211-J200 strain was able to produce lipase (C14) and cystine arylamidase.

### 2.2. Bacterial Identification

**Figure 2:**
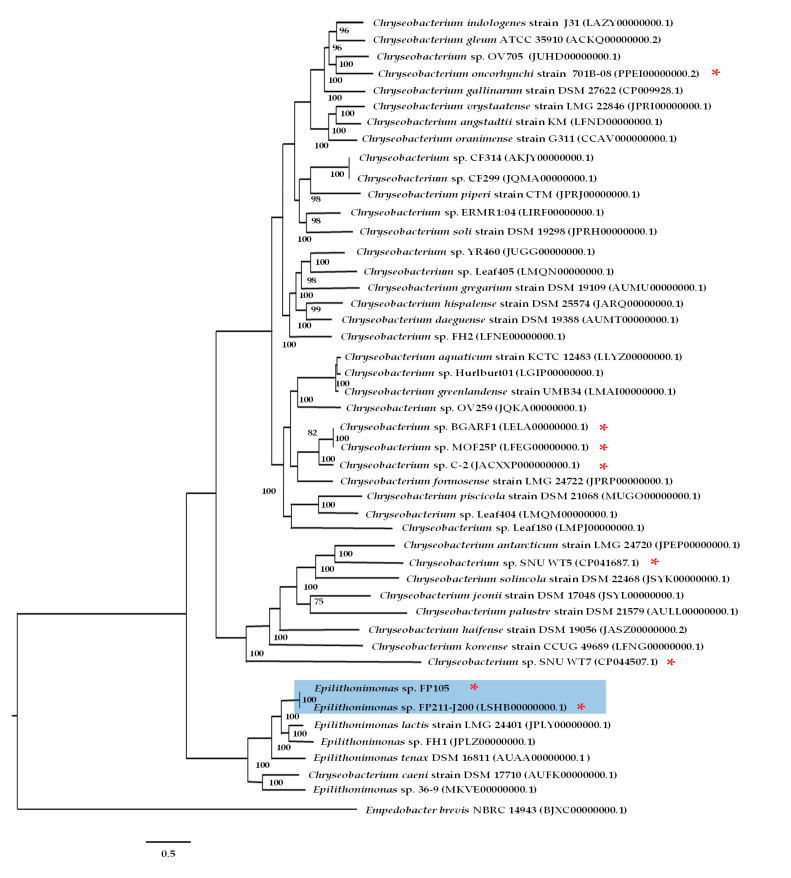
Phylogenetic tree based on the whole genome sequences, showing the relationship between the Chilean *Epilithonimonas* strains (FP105 and FP211-J200) and closely related taxa within the *Chryseobacterium* clade. Tree was constructed using the patric server (https://patricbrc.org/app/PhylogeneticTree, accessed on 12 July 2021). A total of 100 single-copy genes found for 46 genomes and both amino acid and nucleotide sequences were used for each gene. *Empedobacter brevis* NBRC 14943 was used as an outgroup. Accession numbers of each sequence are shown in parentheses. Red asterisks (*) are included to highlight genomes harboring the *tet*(X) gene.

The whole genome sequences of FP105 and FP211-J200 strains were compared with several whole genome sequences currently available for the related members of the *Chryseobacterium* clade. The results are presented as a phylogenetic dendrogram, as is depicted in Figure 2 showing that both Chilean strains are members of the genus *Epilithonimonas*, observing that Chilean strains are genetically most closely related to the *Epilithonimonas lactis* strain LMG24401 isolated from raw cow’s milk in Israel [20].

### 2.3. Minimum Inhibitory Concentrations (MICs)

Both the MIC values of oxytetracycline of FP105 and FP211-J200 strains were 128 µg/mL, whereas reference strains *Escherichia coli* (*E. coli*) ATCC 25922 and *Aeromonas salmonicida* ATCC 33658 used for quality controls exhibited MIC values of oxytetracycline of 0.5 and 0.25 µg/mL, respectively, in agreement with the values recommended by CLSI [21].

### 2.4. Molecular Analysis of tet(X)

When the publicly available genomes of other genera belonging to the Flavobacteriaceae family were investigated, among the genomes of strains deposited in GenBank belonging to *Flavobacterium*, *Chryseobacterium*, and *Empedobacter* genus. They were isolated from fishes, and only three genomes of *Flavobacterium* spp. and six genomes of *Chryseobacterium* spp. were found to harbor the *tet*(X) gene.

Tet(X) was detected in contigs 80 (5749 bp) and 54 (5763 bp) of FP211-J200 and FP105 genomes, respectively, and were identical at nucleotide and amino acid sequence level (identity of 100%) to each other. As shown in Table 1, the amino acid sequence identity of Tet(X) protein produced by *Epilithonimonas* strains exhibited a 100% identity with those synthesized by the *Chryseobacterium* strains (SNU WT5 and SNU WT7) from South Korea [22,23]. Tet(X) proteins from Chilean strains showed an approximately 84% identity with the Tet(X) of *Chryseobacterium* from the UK (MOF25P and BGARF1) [16], Turkey (C2), and Spain (701B-08) [24,25], *Flvobacterium kayseriense* from Turkey (F-47 and F-380) [26], and *Flavobacterium plurextorum* from Spain (CCUG 60112) [27,28]. All of these strains were isolated from fish, and their whole genomes are currently included in the GenBank database (Table 2).

**Figure 3:**
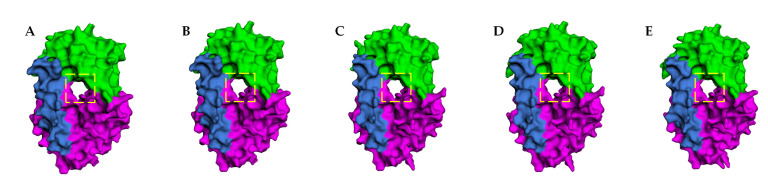
Structural comparison of Tet(X) proteins synthesized by *Bacteroides fragilis* (**A**), *Epilithonimonas* sp. FP105 and FP211-J200 and *Chryseobacterium* sp. SNU WT5 and SNUWT7 (**B**), *C.* sp. MOF25P and BGARF1B (**C**), strains *Flavobacterium kayseriense* F-47 and F-380 (**D**), and strain *F. plurextorum* CCUG 60,112 (**E**) strains isolated from fishes. The substrate-binding domain (light green), FAD-binding domain (pink), and C-terminal helix (blue) are displayed, while the open substrate-loading channel is marked by a yellow dotted box.

As observed in Figure 3, the surface structure of Tet(X) protein first described in *Bacteroides fragilis* (Figure 3A) and used as the structure reference (control) showed a high similarity with Tet(X) proteins synthesized by the Chilean *Epilithonimonas* strains and *Chryseobacterium* strains from South Korea (Figure 3B). However, they showed an important number of differences with the Tet(X) protein structures produced by the *Chryseobacterium* strains from the UK (Figure 3C) and *Flavobacterium* strains from Turkey (Figure 3D) and Spain (Figure 3E) at the substrate binding domain, FAD (flavin adenine dinucleotide)-binding domain, and helix bridge.

The above is mostly explained, because Tet(X) proteins synthesized by the Chilean *Epilithonimonas* and South Korean *Chryseobacterium* strains exhibited only two amino acid substitutions with the sequence of Tet(X) protein produced by *B. fragilis* (Figure S1, Supplementary Data). In contrast, there were a number of amino acid substitutions (64 or 67) between the sequences of the Tet(X) protein synthesized by the *B. gracilis* strain, and the sequences of the Tet(X) proteins synthesized by the *Chryseobacterium* strains from the UK, Turkey, and Spain and the *Flavobacterium* strains (Figure S1, Supplementary Data).

Otherwise, the alignment of the amino acid sequences of Tet(X) from the studied strains did not shown substitutions in the amino acid sequences of the putative substrate-loading cavity composed of FAD-interactive residues and tetracycline-binding residues, thus not affecting their antimicrobial inactivation activity (Figure S1, Supplementary Data).

**Figure 4:**
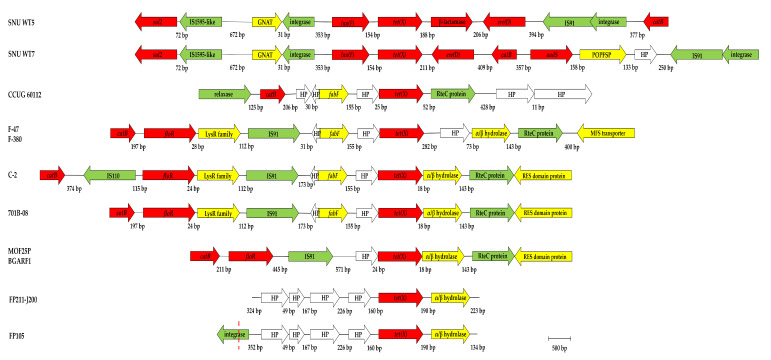
Comparison of the genetic environment of the *tet*(X) genes carried by Chilean *Epilithonimonas* strains (FP105 and FP211-J200) with the available *tet*(X) genes carried by *Chryseobacterium* and *Flavobacterium* strains isolated from fishes. Numbers between ORFs indicate the size of the intergenic region in base pairs (bp). The sequences used in the scheme were those included the GenBank under the accession numbers CP041687.1 (*C.* sp. SNU WT5), CP044507.1 (*C.* sp. SNU WT7), LFEG01000086.1 (*C.* sp. MOF25P), LELA01000055.1 (*C.* sp. BGARF1), JACXXP010000006.1 (*C.* sp. C-2), PPEI02000003.1 (*C. oncorhynchi* 701B-08), JACRUI010000001.1 (*F. kayseriense* F-47), JACRUJ010000001.1 (*F. kayseriense* F-380), MUHD01000006.1 (*F. plurextorum* CCUG 60112) JAHTWS010000054.1 (*E.* sp. FP105), and LSHB01000079.1 (*E.* sp. FP211-J200). Resistome-related genes are in red. Mobilome-related genes are in green. Hypothetical proteins (HP) are in white. Other genes are in yellow.

The genetic environment of *tet*(X) genes of *Epilithonimonas* sp. FP105 and FP211-J200 strains was characterized by the upstream location of a sequence encoding an hypothetical protein, whereas the downstream of the *tet*(X) gene was detected by a gene encoding an alpha/beta hydrolase (Figure 4). When the neighborhood of the *tet*(X) genes carried by both Chilean *Epilithonimonas* strains was compared to the other fish *Flavobacteriaceae tet*(X), the genetic surroundings of *tet*(X) genes carried by the *Epilithonimonas* strains were very different to those of the South Korean *Chryseobacterium* strains. The *Chryseobacterium* strains carried a *lnu*(F) gene encoding a lincosamide nucleotidyltransferase protein at the upstream location and a downstream location of a gene encoding a class D β–lactamase (SNU WT5). While both carried the *ere(D)* gene, they were responsible for erythromycin resistance (SNU WT7) found in different locations (Figure 4).

The same upstream and downstream flanking genes of *tet*(X) genes carried by the Chilean strains were observed in the *tet*(X) genes carried by the *Chryseobacterium* spp. strains from UK, Turkey, and Spain. While the *tet*(X) genes carried by the *Flavobacterium* strains also exhibited an upstream flanking gene encoding an hypothetical protein, they had different downstream flanking genes (Figure 4).

The Chilean strains showed a very low amino acid identity to the flanking hypothetical proteins detected in the *Chryseobacterium*, *Flavobacterium kayseiense* (*F. kayseriense*; 26.51%), and *Flavobacterium plurextorum* (25.30%) strains. Otherwise, the genes encoding the hypothetical proteins carried by the *Chryseobacterium* and *Flavobacterium* strains had an amino acid identity of more than 90% (Table S1, Supplementary Data).

As is shown in Figure 4, four *Chryseobacterium* spp. (MOF25P, BGARF1, C-2, and 701B-08) and two *F. kayseriense* (F-47 and F-380) strains also carried a downstream located gene encoding an alpha/beta hydrolase (Figure 3). However, Chilean strains showed a very low amino acid identity to the flanking alpha/beta hydrolase sequences detected in the *Chryseobacterium* (27.13%) and *F. kayseriense* (27.67%) strains. All of the hydrolase genes carried by the *Chryseobacterium* strains shared a 100% amino acid identity between themselves, as was observed between the same genes carried by the *F. kayseriense* strains (Table S2, Supplementary Data).

## 3. Discussion

### 3.1. Bacterial Identification

Many representatives belonging to the Flavobacteriaceae family have a very relevant role as fish pathogens, as was exhaustively described [29,30]. The genus Chryseobacterium has been frequently isolated as the causative agent of disease ocurring in freshwater fish and particularly rainbow trout diseases, including *C. viscerum, C. oncorhynchae, C. tructae, C.* *shigense, C. chaponense, C. piscicola* species [24,31,32]. Other members of the Flavobacteriaceae family have been previously isolated from diseased farmed salmonids in Chile [33,34,35].

It is not uncommon to misidentify bacteria as *F.* *psychrophilum* as occurs with the studied strains, which could be due to the fact that they be correctly identified as Epilithonimonas sp., a member of the Chryseobacterium clade, shown in this study [36,37].

This misidentification is very relevant considering that *F. psychrophilum* species does not carry specific genes for resistance to tetracyclines, as occurs with pathogenic strains of fish and humans belonging to the Chryseobacterium clade, including several species belonging to the Chryseobacterium, Empedobacter, and Riemerella genus, among others, which have been reported to carry the *tet*(X) gene [38,39,40,41,42]. As a result of this, it has been hypothesized that the Flavobacteriaceae family could be a potential ancestral source of the tigecycline-resistance gene *tet*(X), as was recently claimed [43]. Thus, the detection of genes encoding for tetracycline resistance in fish pathogenic bacteria is of concern for the sustainability of this industry.

### 3.2. Detection of tet(X) Genes

It has been demonstrated that using culture-independent methods, water samples taken from fish farms with recent oxytetracycline use have significantly higher frequencies of *tet* genes than water from farms without recent oxytetracycline therapies exceeding by more than two-fold that of untreated farms [44].

Furthermore, the occurrence of tetracycline-resistance encoding genes in aquaculture-impacted environments is frequent, as was observed in studies showing that 57.14% of the total resistant bacteria recover from aquaculture environments [45]. All 108 resistant strains isolated from aquaculture ponds in China were positive for a *tet* gene [46], which persisted at aquaculture farms even in the absence of a selection pressure [47]. Xiong et al. [48] found a high relative abundance (10^−5^ to 10^−3^ of gene copies/16S ribosomal RNA (rRNA) gene copies) of various *tet* genes, including *tet*(X) in sediment samples from fish ponds without antimicrobial usage. They concluded that detected antimicrobials, such as oxytetracycline and doxycycline, were introduced by applied organic wastes from terrestrial animals. The authors suggested that sediments are the main reservoirs of tetracycline resistance genes in aquaculture environments in China. Another study has shown that farmed fish feces are a relevant source of tetracycline resistance genes in the farm sediments, despite the absence of antibiotic treatments at the studied farms [49].

In a recent article [50], the authors performed a whole-genome sequencing analysis of the *Chryseobacterium aquaticum* strain C-174, isolated from diseased farmed rainbow trout in Turkey, reporting this strain carries many tetracycline-resistance genes, including *tet*(32), *tet*(60), *tet*(T), *tet*(X), and *tet*(W). However, when the C-174 genome was further analyzed, we confirmed that all of these genes, and other reported antimicrobial-resistance genes were misidentified, not corresponding to antimicrobial-resistance genes. The *tet*(X) sequence (NMR 36027.1) has only a 28% identity with the *tet*(X) sequence included in the GenBank database, whereas it has a 99% identity with other flavin-dependent monooxygenases which do not belong to the Tet(X) group (WP_050378416.1).

It must be noted that among all *tet*(X) genes reported in flavobacteriaceae strains from fish, only in this study, it was confirmed that the detected *tet*(X) genes conferred the tetracycline-resistance phenotype, considering that both *Epilithonimonas* strains showed an MIC of oxytetracycline of 128 µg/mL, which is within the expected level of resistance to oxytetracycline mediated by the Tet(X) activity. This value is in agreement with previous studies in which *tet*(X) genes heterologously expressed by *E. coli* transconjugants harboring recombinant plasmids exhibited MIC values of 128–256 µg/mL [12,40,51,52].

### 3.3. Molecular Analysis of tet(X)

The *tet*(X) genes carried by the Chilean strains were 99.83% identcal to the wild-type (WT) *tet*(X) gene, which was first recovered in *Bacteroides fragilis* [53], and only two mutations (A280G and G1077C) were detected in the *tet*(X) genes carried by the Chilean *Epilithonimonas* strains resulting in amino acid substitutions at the corresponding sites (K94E and M359I), (Figure S1). These two mutatios were previously reported in a *tet*(X) variant carried by an *Empedobacter falsenii* strain isolated from a chinese patient [41]. The authors demonstrated that both amino acids are located far from the active site regions, thus not affecting the activity of this Tet(X) protein.

Important differences in the genetic neighborhood of *tet*(X) genes carried by the Chilean strains, when compared to the other *tet*(X)-carrying strains isolated from fishes, were observed. It must be noted that all *Chryseobacterium* and *Flavobacterium* strains analyzed in the study carried the *catB* gene, encoding for a chloramphenicol acetyltransferase in the *tet*(X) neighborhood, whereas this gene was absent in the genome of Chilean *Epilithonimonas* strains.

In addition, the insertion sequence IS91 was detected in the genetic environment of the majority of the analyzed strains, with the only exception of the Chilean strains and *F. plurextorum*. In addition, the *tet*(X) gene in *Bacteroides fragilis* was found to be inserted in the transposon Tn4400, with an upstream location of the erythromycin-resistance gene *ermF* [53], very different from what was observed in the strains in this study.

This is the first report of a *tet*(X) gene detected in pathogenic species belonging to the Flavobacteriaceae family in Chilean aquaculture and prompts the necessity to investigate the carriage of this gene by bacteria associated to Chilean salmonid farms and farmed salmonid microbiota, considering that mobilome elements such as ISCR2, IS26, and many conjugative and mobilizable plasmids could play an essential role in the acquisition and dissemination of *tet*(X) genes in natural reservoirs [54]. Furthermore, the potential role of pathogenic strains belonging to the Flavobacteriales occurring in Chilean salmonid farms as reservoirs of *tet*(X) genes must be elucidated.

## 4. Materials and Methods

### 4.1. Bacterial Strains

The bacterial strains FP105 and FP233-J200 isolated from fin lesion (FP105) and kidney (FP223-J200) of diseased rainbow trout *Oncorhynchus mykiss* positively diagnosed with Flavobacteriosis sampled from two freshwater Chilean farms located in the South of Chile (Llanquihue Lake and Cude River, respectively) were studied. Strains were isolated in the fish pathological diagnostic laboratory ADL Diagnostics and sent to the Aquatic Pathobiology Lab of the Universidad Católica del Norte. The strains were purified using Tryptone-Yeast Extract Salt (TYES) agar [55] and stored at −85 °C in CryoBank^TM^ vials (Mast Diagnostica, Reinfeld, Germany). Strains were grown in TYES agar at 25 °C for 24 h prior to use (Figure 3).

### 4.2. Phenotypic Characterization

The phenotypic tests of Gram staining, cell morphology, colony morphology grown onto TYES agar, and oxidation/fermentation (O/F) of glucose were determined according to the procedures described in Buller [56]. Furthermore, several key characteristics for the description of bacterial strains belonging to the *Flavobacteriaceae* family [57], such as production of oxidase and catalase, hydrolysis of aesculin and gelatin, reduction of nitrate to nitrite, indole production, production of flexirubin-type pigment, growth at 25 °C on R2A (Becton-Dickinson, Sparks, MD, USA), MacConkey (Becton-Dickinson, Sparks, MD, USA), Trypticase Soy (TSA, Becton-Dickinson, Sparks, MD, USA) agar, and in Brain Heart Infusion (BHI, Becton-Dickinson, Sparks, MD, USA) broth added with 1.0, 2.0, 3.0, 4.5, and 6.5% NaCl were performed using procedures as previously described [56,58]. In addition, growth at 15, 25, 30, 37, and 42 °C in a BHI broth was assayed.

Other enzymatic activities of FP105 and FP223-J200 strains were determined using the API ZYM system (bioMérieux, Marcy-l’Etoile, France) according to the manufacturer’s guidelines. Test strips were read after 5 min as indicated by the manufacturer, and each assay was performed twice to ensure reproducibility.

### 4.3. Bacterial DNA Extraction and Sequencing

The genomic DNA of strains was extracted and purified using the commercial Wizard^®^ Genomic DNA Purification kit (Promega, Madison, WI, USA), following the instructions of the supplier. The whole genomic DNA was sequenced by Macrogen USA (Rockville, MD, USA) using the Illumina MiSeq platform, and 500-bp inserts from paired-end sequencing were utilized in the genomic library. Low-quality reads were trimmed with a quality threshold of Q20; the trimmed reads were then subjected to de novo assembly using the SPAdes assembler [59]. The reads were assembled to 83 (FP211-J200) and 63 (FP105) scaffolds with the 4,110,772 bp and 4,124,333 bp total genomes lengths for each strain, respectively. Genome was annotated using the NCBI Prokaryotic Genome Annotation Pipeline (PGAP) service. In total, 3748 and 3778 coding sequences of strains FP211-J200 and FP105, respectively, were annotated in the NCBI database. The GenBank accession number of the complete genome sequence of FP211-J200 strain is LSHB01000000 [19], and the genome sequence of FP105 strain was registered under the GenBank accession number of JAHTWS000000000.1.

### 4.4. Bacterial Identification

The whole genomic DNA sequences were used to identify the strains by a genome comparison using the PATRIC bioinformatics platform. FP105 and FP211-J200 strains were identified by comparison analysis of 100 single copy genes using the PATRIC server (https://patricbrc.org/app/PhylogeneticTree, accessed on 10 April 2021). As described in PATRIC platform, protein sequences were aligned using MUSCLE, and the nucleotide coding gene sequences were aligned using the Codon_align function of BioPython. A concatenated alignment of all proteins and nucleotides were written to a phylip formatted file, and then a partitions file for RaxML is generated, describing the alignment in terms of the proteins and then the first, second and third codon positions. Support values were generated using 100 rounds of the “Rapid” bootstrapping option of RaxML.

The whole genome sequences of FP105 and FP211-J200 strains were compared with a total of 46 whole genome sequences currently available for related members of the *Chryseobacterium* clade.

### 4.5. MICs

The MICs of oxytetracycline of FP105 and FP211-J200 isolates were determined by a microdilution procedure, as recommended by the CLSI guideline M07-A10 [60] and previously described [61]. Conical bottom microplates added with a cation-adjusted Mueller–Hinton broth (Difco Labs, NJ, USA) were inoculated with the antibiotic to obtain final series of two-fold concentrations in the range of 0.0625–512 µg/mL, and bacterial suspensions were inoculated in triplicate microplates, delivering approximately 10^4^ colony-forming units per well and incubated at 28 °C for 24 h. The reference strains *E. coli* ATCC 25,922 and *Aeromonas salmonicida* ATCC 33,658 were included as quality controls, as was recommended [21]. All assays were performed twice to check the reproducibility of the assay.

### 4.6. Molecular Analysis of tet(X) Genes

The analysis of the *tet*(X) gene sequences and their genetic environments were performed using the contigs derived from genomic sequencing using the BioEdit 7.2.5 software [62] and subsequent comparison by BLAST computational analysis with the sequences of *tet*(X) genes carried by *Chryseobacterium* strains isolated from fishes included in the GenBank database and/or previously reported [16,25].

The modelling of Tet(X) proteins produced by the studied strains was based on published Tet(X) from *Bacteroides thetaiotaomicron* (PDB accession number: c2xdoC) using the online server Phyre2 [63]. The substrate-binding domain (light green), the FAD-binding domain (pink), and the C-terminal helix (blue) were displayed, while the open substrate-loading channel was marked in a yellow dotted box. In addition, the alignment of the amino acid sequences of Tet(X) proteins produced by the Chilean *Epilithonimonas* strains with the sequences of the other Tet(X) variants found in Flavobacteriaceae from fishes were conducted with Clustal Omega (https://www.ebi.ac.uk/Tools/msa/clustalo/, accessed on 15 June 2021) generating its output with ESPript 3.0 (http://espript.ibcp.fr/ESPript/cgi-bin/ESPript.cgi, accessed on 15 June 2021) [64]. A secondary structure based on the Tet(X) protein detected in *Bacteroides fragilis* (PDB number: 4A6N) served as a structure reference.

## 5. Conclusions

In conclusion, the results of this study demonstrated, for the first time, the carriage of the *tet*(X) gene by bacterial strains isolated from reared rainbow trout affected with Flavobacteriosis in Chilean freshwater salmonid farming and the carriage of this gene by the *Epilithonimonas* genus. The detection of the *tet*(X) gene in these representatives of the *Chryseobacterium* clade reinforces the hypothesis that this taxonomic group may serve as an important environmental reservoir of this gene. Furthermore, the genetic environment of *tet*(X) carried by the *Epilithonimonas* strains is very different to those detected in two *Chryseobacterium* isolates recovered from fish in South Korea, despite their high amino acid similarity, suggesting the need to gain knowledge of the genetic epidemiology of *tet*(X) genes carried by fish pathogenic bacteria. Finally, this study demonstrates the carriage of *tet*(X) genes by two pathogenic bacteria from reared rainbow trout in Chile may become a threat due to the frequent oxytetracycline-based treatment of Flavobacteriosis in Chile. Finally, this study demonstrated the carriage of *tet*(X) genes by two pathogenic bacteria isolated from farmed rainbow trout in Chile, which may become a threat to the Chilean industry due to the frequent use of oxytetracycline for the treatment of Flavobacteriosis.

## Figures and Tables

**Table 1:** Enzymatic properties of the *Epilithonimonas* FP105 and FP211-J200 strains by using the API ZYM system (BioMerieux).

Enzyme	Activity	
	FP105	FP211-J200
Control	Negative	Negative
Alkaline phosphatase	Positive	Positive
Esterase (C4)	Positive	Positive
Esterase lipase (C8)	Positive	Positive
Lipase (C14)	Negative	Positive
Leucine arylamidase	Positive	Positive
Valine arylamidase	Positive	Positive
Cystine arylamidase	Negative	Positive
Trypsin	Negative	Negative
α-chymotrypsin	Negative	Negative
Acid phosphatase	Positive	Positive
Naphthol-AS-BI-phosphohydrolase	Positive	Positive
α-galactosidase	Negative	Negative
β-galactosidase	Negative	Negative
β-glucoronidase	Negative	Negative
α-glucosidase	Negative	Negative
β-glucosidase	Negative	Negative
*N*-acetyl-β-glucosaminidase	Negative	Negative
α-mannosidase	Negative	Negative
α-fucosidase	Negative	Negative

**Table 2:** Similarity of nucleotide and amino acid sequences of Tet(X) synthesized by *Flavobacteriaceae* strains isolated from fishes and Tet(X) produced by *Bacteroides fragilis* (control).

Strain	Percentage of Nucleotide/Amino Acid Similarity * (%)											
	Control	FP105	FP211-J200	SNU WT5	SNU WT7	MOF25P	BGARF1	C2	701B-08	F-47	F-380	CCGU
Control	100/100	99.83/99.48	99.83/99.48	99.83/99.48	99.83/99.48	86.00/83.20	86.00/83.20	85.91/83.20	85.82/83.20	83.59/82.69	83.59/82.69	83.76/83.20
FP105		100/100	100/100	100/100	100/100	87.25/84.66	87.25/84.66	87.16/84.66	87.07/84.66	84.78/84.13	84.78/84.13	84.96/84.66
FP211-J200			100/100	100/100	100/100	87.25/84.66	87.25/84.66	87.16/84.66	87.07/84.66	84.78/84.13	84.78/84.13	84.96/84.66
SNU WT5				100/100	100/100	87.25/84.66	87.25/84.66	87.16/84.66	87.07/84.66	84.78/84.13	84.78/84.13	84.96/84.66
SNU WT7					100/100	87.25/84.66	87.25/84.66	87.16/84.66	87.07/84.66	84.78/84.13	84.78/84.13	84.96/84.66
MOF25P						100/100	100/100	99.91/100	99.82/100	92.61/93.92	92.61/93.92	90.85/91.27
BGARF1							100/100	99.91/100	99.82/100	92.61/93.92	92.61/93.92	90.85/91.27
C-2								100/100	99.91/100	92.52/93.92	92.52/93.92	90.77/91.27
701B-08									100/100	92.44/93.92	92.44/93.92	90.68/91.27
F-47										100/100	100/100	92.00/92.06
F-380											100/100	92.00/92.06
CCUG 60112												100/100

* control (*B. fragilis*), *tet*(X), M37699.1; Tet(X), AAA27471.1; *Epilithonimonas* sp. FP105, *tet*(X), JAHTWS010000054.1; Tet(X), MBV6881964.1; *Epilithonimonas* sp. FP211-J200, *tet*(X), LSH01000079.1; Tet(X), OAH64793.1; *Chryseobacterium* sp. SNU WT5, *tet*(X), CP041687.1; Tet(X), QDP85680.1; *Chryseobacterium* sp., SNU WT7 *tet*(X), CP044507.1; Tet(X), QFG52251.1; *Chryseobacterium* sp. MOF25P, *tet*(X), LFEG01000086.1; Tet(X), OBW41066.1; *Chryseobacterium* sp. BGARF1, *tet*(X), LELA01000055.1; Tet(X), OBW45804.1; *Chryseobacterium* sp. C-2, *tet*(X), JACXXP010000006.1; Tet(X), MBD3904424.1; *Chryseobacterium oncorhynchi* 701B-08, *tet*(X), PPEI02000003.1; Tet(X), PWN64762.1. *Flavobacterium kayseriense* F-47, *tet*(X), JACRUI010000001.1; Tet(X), MBC5847276.1. *F. kayseriense* F-380, *tet*(X), JACRUJ010000001.1; Tet(X), MBC5840054.1. *Flavobacterium plurextorum* CCUG 60112, *tet*(X), MUHD01000006.1; Tet(X), OXB10245.1.

## Data Availability

The whole-genome sequences of FP105 and FP211-J200 strains have been deposited at DDBJ/ENA/GenBank under the accession numbers of JAHTWS000000000.1 and LSHB01000000.1, respectively.

## Supplementary Materials

The following are available online at https://www.mdpi.com/article/10.3390/antibiotics10091051/s1, Figure S1: Alignment of the amino acid sequences of Tet(X) proteins produced by the Chilean Epilithonimonas strains with the sequences of the other Tet(X) variants found in Flavobacteriaceae from fishes, Table S1: Similarity of nucleotide and amino acid sequences of alpha/beta hydrolase flanking the *tet*(X) gene carried by strains belonging to the Flavobacteriaceae family isolated from fishes, Table S2: Similarity of nucleotide and amino acid sequences of hypothetical protein flanking the *tet*(X) gene carried by strains belonging to the Flavobacteriaceae family isolated from fishes.
