# Supplementary material for: Genetic Characterization of the Tetracycline-Resistance Gene tet(X) Carried by Two Epilithonimonas Strains Isolated from Farmed Diseased Rainbow Trout, Oncorhynchus mykiss in Chile
Source: Antibiotics (Basel). 2021 Aug 29;10(9):1051. doi: 10.3390/antibiotics10091051 (PMC8464911; doi:10.3390/antibiotics10091051)
Supplement: Supplementary file 1 [file antibiotics-10-01051-s001.zip › Figure S1.pdf]

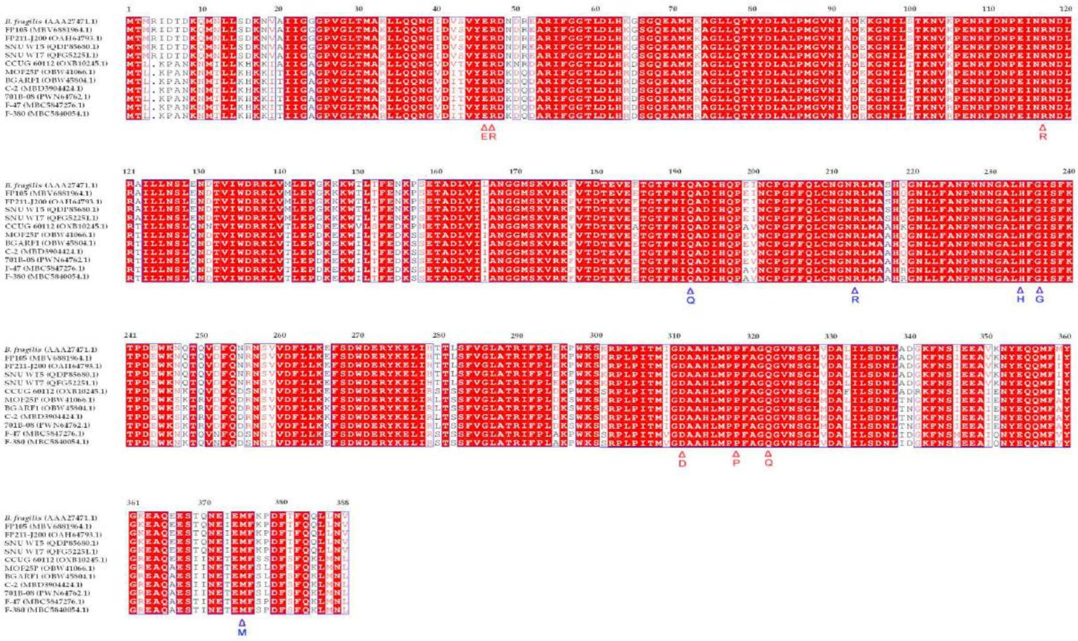

Figure S1. Alignment of the amino acid sequences of Tet(X) proteins produced by the Chilean *Epilithonimonas* strains with the sequences of the other Tet(X) variants found in Flavobacteriaceae from fishes. Multiple sequence alignment was conducted with Clustal Omega (https://www.ebi.ac.uk/Tools/msa/clustalo/), generating its output with ESPript 3.0 (http://espript.ibcp.fr/ESPript/cgi-bin/ESPript.cgi). A secondary structure based on the Tet(X) protein detected in *Bacteroides fragilis* served as the structure reference. Identical residues are in white letters with red background, and different residues are black letters with white background. The putative substrateloading cavity is composed of FAD-interactive residues (red triangles) and Tigecycline-binding residues (blue triangles).
