# Supplementary material for: Genetic Characterization of the Tetracycline-Resistance Gene tet(X) Carried by Two Epilithonimonas Strains Isolated from Farmed Diseased Rainbow Trout, Oncorhynchus mykiss in Chile
Source: Antibiotics (Basel). 2021 Aug 29;10(9):1051. doi: 10.3390/antibiotics10091051 (PMC8464911; doi:10.3390/antibiotics10091051)
Supplement: Supplementary file 1 [file antibiotics-10-01051-s001.zip › Table S1.pdf]

Table S1. Similarity of nucleotide and amino acid sequences of alpha/beta hydrolase flanking the *tet*(X) gene carried by strains belonging to the *Flavobacteriaceae* family isolated from fishes.

Strain	Percentage of Nucleotide/Amino acid similarity* (%)							
	FP105	FP211-J200	MOF25P	BGARF1	C2	701B-08	F-47	F-380
Epilithonimonas sp. FP105	100/100	100/100	48.12/27.13	48.12/27.13	48.12/27.13	48.12/27.13	47.62/27.67	47.62/27.67
Epilithonimonas sp. FP211-J200		100/100	48.12/27.13	48.12/27.13	48.12/27.13	48.12/27.13	47.62/27.67	47.62/27.67
Chryseobacterium sp. MOF25P			100/100	100/100	100/100	100/100	88.39/90.21	88.39/90.21
Chryseobacterium sp. BGARF1				100/100	100/100	100/100	88.39/90.21	88.39/90.21
Chryseobacterium sp. C-2					100/100	100/100	88.39/90.21	88.39/90.21
Chryseobacterium oncorhynchi 701B-08						100/100	88.39/90.21	88.39/90.21
Flavobacterium kayseriense F-47							100/100	100/100
Flavobacterium kayseriense F-380								100/100

*: The accession numbers of genes (contigs) and proteins are as follows: JAHTWS010000054.1 and MBV6881965.1 from *E.* sp. FP105; LSHB01000079.1 and OAH64794.1 from *E.* sp. FP211-J200.1; LFEG01000086.1 and OBW41065.1 from *C.* sp. MOF25P; LELA01000055.1 and OBW45805.1 from *C.* sp. BGARF1; JACXXP010000006.1 and MBD3904423.1 from *C.* sp. C-2; PPEI02000003.1 and PWN64761.1 from *C. oncorhynchi* 701B-08; JACRUJ010000001.1 and MBC5840052.1from *F. kayseriense* F-380; JACRUI010000001.1 and MBC5847278.1 from *F. kayseriense* F-47.
