# Supplementary material for: Genetic Characterization of the Tetracycline-Resistance Gene tet(X) Carried by Two Epilithonimonas Strains Isolated from Farmed Diseased Rainbow Trout, Oncorhynchus mykiss in Chile
Source: Antibiotics (Basel). 2021 Aug 29;10(9):1051. doi: 10.3390/antibiotics10091051 (PMC8464911; doi:10.3390/antibiotics10091051)
Supplement: Supplementary file 1 [file antibiotics-10-01051-s001.zip › Table S2.pdf]

Table S2. Similarity of nucleotide and amino acid sequences of hypothetical protein flanking the *tet*(X) gene carried by strains belonging to the *Flavobacteriaceae* family isolated from fishes.

Strain	Percentage of Nucleotide/Amino acid similarity* (%)								
	FP105	FP211-J200	MOF25P	BGARF1	C2	701B-08	F-47	F-380	CCUG
Epilithonimonas sp. FP105	100/100	100/100	51.25/26.51	51.25/26.51	51.25/26.51	51.25/26.51	48.22/26.51	48.22/26.51	48.77/25.30
Epilithonimonas sp. FP211-J200		100/100	51.25/26.51	51.25/26.51	51.25/26.51	51.25/26.51	48.22/26.51	48.22/26.51	48.77/25.30
Chryseobacterium sp. MOF25P			100/100	100/100	99.74/99.20	99.74/99.20	100/100	100/100	89.68/91.20
Chryseobacterium sp. BGARF1				100/100	99.74/99.20	99.74/99.20	100/100	100/100	89.68/91.20
Chryseobacterium sp. C-2					100/100	100/100	99.74/99.20	99.74/99.20	89.42/90.40
Chryseobacterium oncorhynchi 701B-08						100/100	99.74/99.20	99.74/99.20	89.42/90.40
Flavobacterium kayseriense F-47							100/100	100/100	89.68/91.20
Flavobacterium kayseriense F-380								100/100	89.68/91.20
Flavobacterium plurextorum CCUG 60112F									100/100

*: The accession numbers of genes (contigs) and proteins are as follows: JAHTWS010000054.1 and MBV6881963.1 from *E.* sp. FP105; LSHB01000079.1 and OAH64792.1 from *E.* sp. FP211-J200; LFEG01000086.1 and OBW41067.1 from *C.* sp. MOF25P; LELA01000055.1 and OBW45803.1 from *C.* sp. BGARF1; JACXXP010000006.1 and MBD3904425.1 from *C.* sp. C-2; PPEI02000003.1 and PWN64763.1 from *C. oncorhynchi* 701B-08; JACRUI010000001.1 and MBC5847275.1 from *F. kayseriense* F-47; JACRUJ010000001.1 and MBC5840055.1 from *F. kayseriense* F-380; MUHD01000006.1 and OXB10244.1 from *F. plurextorum* CCUG 60112.
